# Notes from past Meetings of the Lippe Society

**Published:** 1890-02

**Authors:** 


### NOTES FROM PAST MEETINGS OF THE LIPPE SOCIETY.

Dr. Lippe said that Phytolacca was indicated in nursing sore mouth.

Dr. Allen had had a case of sciatica cured at once by Carbo-veg. 16 M.

Dr. Lippe said that Arsenic was the best remedy to stop the ravages of phagedenic chancre.

Dr. Geo. H. Clark had cured a case of lupus complicated with chancre by Lycopodium.

Dr. Allen had cured a case of epithelioma by Lycopodium very high.

Dr. Lippe had been very successful in controlling a case of typhus fever with Bryonia, one dose.

Dr. Guernsey had treated a case of difficult respiration, especially in the act of expiration. Mephitis was the remedy, and it acted with excellent effect.

Dr. Lippe spoke of valvular heart disease, and said he had had excellent results in prescribing old Madeira wine for such cases.

Dr. Mahlon Preston reported a case of a woman who passed no urine for three months!

Dr. Allen had had a similar case.

Dr. Lippe said that in cases of double vision with one object higher than the other, Stramon. was apt to be the remedy.

Dr. Guernsey has cured a case of syphlitic sore mouth, having as a principal indication *burning and stinging*, with Apis.

Dr. Allen had a case of diarrhœa with vomiting and prostration. He gave Veratrum with relief; but spasms and cramps of the limbs continued. Finally he observed that the patient would remain *uncovered*, and was very restless. Secale cornut. was given and it cured at once.

Dr. Lippe said that he had that day observed tan spread before a house where was a very sick patient. He at once thought of Nitric-acid as the probable remedy for the case. Nitric acid has great sensitiveness to noise and jarring.

Dr. Guernsey said that Gelseminum has headache commencing in the nape of the neck and going all over the head to the forehead.

Dr. Lippe said that Belladonna and Calcarea-carb. have the same symptom.

Dr. Fellger said that Argent-nitricum has the same symptom.

Dr. Allen had a case of probable frost-bite. There was swelling of the great toe with severe pain. Also severe pain as of a sharp instrument passing upward between the metatarsal bones and feeling of a piece of ice lying there. Berberis relieved the pain and cured the whole condition.

A patient had a sense of something warm rising into the throat, causing cough. Zinc was the remedy.

Dr. Lippe said that in whatever disease a patient may have even bone troubles, if the patient can't bear to be moved or to be touched, China was the remedy.

Dr. Lippe remarked that the great remedy for headache in school girls was Phosphoric acid.

Dr. C. Carleton Smith said he had cured such headaches in school girls with Phosphoric acid.

Dr. Guernsey related the case of a patient with face-ache. The patient complained that the pillow felt as hard as iron. Arnica was given upon this indication, and brought immediate relief.

Dr. Lee said Phosphorus has same symptom. Ferrum magneticum has the symptom that as soon as the patient begins to eat, he must go to stool.

Dr. Allen, said: Kali-bichrom. has sudden desire for stool, but it comes before he has had time to get out of bed.

The Sulphur patient does have time to get out of bed, but he must go quickly.

Dr. Fellger has good results from Lithium-carb. in cases of encysted stone in the bladder.

Dr. Guernsey finds Ledum a good remedy for horses that have wounds from nails. He puts some of the tincture upon a piece of absorbent cotton and applies it to the wound.

Dr. Lippe said: For patients having unreasonable fear of cholera, Lachesis was the remedy.

Dr. Guernsey gave an account of a woman with puerperal convulsions to whom he administered Arnica with the most gratifying results. There were bruises in the face which helped him in the selection of the remedy.

Dr. Guernsey spoke of the peculiar symptom of Moschus: one cheek is red and cold and the other pale and hot.

Dr. Allen had a case of ulceration between the buttocks in a little child. The ulcers were blue and offensive, and surrounded by pimples. The child was fearful of being touched, and cried whenever the doctor approached. Dr. Allen gave Lachesis$^{cm}$, and in a week the child was cured. He had never seen such prompt action from a remedy.

Dr. Allen had a case of paralysis which resisted the remedies. The patient complained of coldness of the patella. This symptom led him to study Aurum, which was given and relieved immediately. The woman was likely to recover completely.

Dr. Lippe relieved a case of paralysis with jumping of the legs by Argentum nitricum.

Dr. Guernsey had been successful in curing canker of the mouth with Antimon-crud. He could not, however, give any reliable indications.

One case had the symptom, cold saliva flows from the mouth. Phytolacca relieved this case.

Dr. Lippe said that Caladium had the same symptom.

